# Comparison of the Jcerity endoscoper airway and the endotracheal tube in endoscopic esophageal variceal ligation: a prospective randomized controlled trial

**DOI:** 10.1038/s41598-023-39086-6

**Published:** 2023-07-22

**Authors:** Le Zhang, Lu Li, Jun Wang, Can Zhao, Erxian Zhao, Yanrong Li, Yunqi Lv

**Affiliations:** 1 Department of Anesthesiology, Pain and Perioperative Medicine, The First Affiliated Hospital of Zhengzhou University, No. 1 Jianshe East Road, Erqi District, Zhengzhou, 450052 Henan China; 2 Department of Operation Room, The First Affiliated Hospital of Zhengzhou University, Zhengzhou, Henan China

**Keywords:** Diseases, Gastroenterology

## Abstract

Various airway techniques have been used in endoscopic esophageal variceal ligation (EVL). In this respect, Jcerity endoscoper airway (JEA) is a novel laryngeal mask airway that is designed for use in gastrointestinal endoscopy. In the present study, 164 patients who underwent EVL were randomly divided into JEA group or endotracheal tube (ETT) group (ratio: 1:1). Success rate of endoscopic procedure, endoscope insertion time, procedure duration, recovery time, airway technique extubation time, anesthesia costs, hospital stay duration, complications, and hemodynamic parameters were recorded. The success rate of EVL in the JEA group was noninferior to that in the ETT group (98.8% vs. 100.0%). The airway insertion time, anesthesia duration, and recovery time were significantly shorter in the JEA group than in the ETT group (*p* < 0.001). Furthermore, the blood pressure during extubation was more stable in the JEA group (*p* < 0.001). Moreover, there were less heart rate variations during intubation (*p* < 0.005) and extubation (*p* < 0.05) in the JEA group. Nonetheless, the endoscopists’ satisfaction scores were comparable between the two groups. Overall, our findings suggest that JEA is efficient and safe for clinical use in EVL.

**Trial registration:** Chinese Clinical Trial Registry, ChiCTR2000031892, Registered April 13, 2020, https://www.chictr.org.cn/searchproj.html.

## Introduction

There has been a rapid increase in the use of nonoperating room anesthesia (NORA) sites and services over the past decade^1^, with the use of anesthesia services in gastrointestinal (GI) suites increasing from 10.8 to 17.3% between 2010 and 2014^2^. Patients in the GI suite frequently receive anesthesia without an endotracheal tube. During tubeless anesthesia, patients frequently transition between different depths of anesthesia^3^. Although sedation is usually performed safely, sedation without a controlled airway has been shown to have a higher incidence of respiratory depression (insufficient ventilation) in patients who are treated in non-ORs, and the resulting incidence of death events is also higher than that of patients who are treated in ORs^4^. Of note, cardiorespiratory arrest and hypoxia occurred more frequently in patients who underwent upper GI procedures than in those who underwent colonoscopy^5^.

EVL is one of the most challenging endoscopic procedures due to its high risks of perioperative bleeding and reflux^6^. Consequently, EVL is usually performed under deep anesthesia with or without endotracheal intubation^7^. Deep anesthesia can reduce body movement, choking, etc., thus reducing the risk of bleeding. Endotracheal intubation has shown great promise in achieving airway protection. It has been reported that patients who are operated on under propofol-based sedation without endotracheal intubation have a higher incidence of sedation-related adverse events (SRAEs) than patients who are operated on under anesthesia with endotracheal intubation (51.5% vs. 9.9%, *p* < 0.001). SRAEs include any peripheral oxygen saturation (SpO2) < 90% and the use of airway maneuvers^8^. Nonetheless, general anesthesia via endotracheal intubation necessitates a substantial amount of anesthetic drugs that can further compromise liver function and prolong recovery. Furthermore, it can lead to significant hemodynamic fluctuations. Thus, different airway techniques have been explored for use in endoscopic EVL to enhance airway management, prevent hypoxia, and circumvent the need for intubation, ultimately improving patient outcomes.

**Figure 1:**
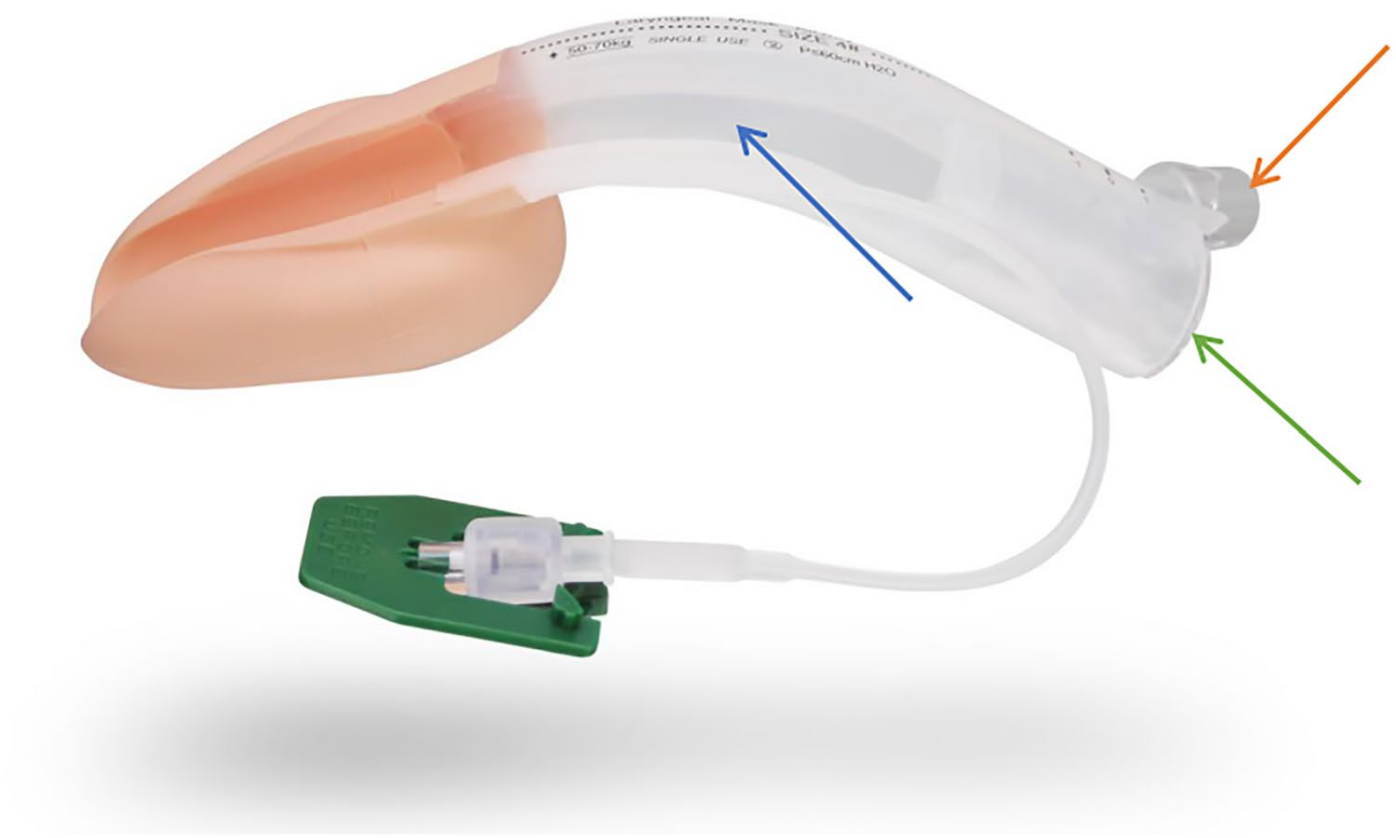
The dual channel Jcerity endoscope airway with silicon airway channel (orange arrow), and a separate endoscopy channel (green arrow) with groove design (blue arrow) for endoscope, which was shot and edited by Lu Li.

The Jcerity Endoscoper Airway (Zhejiang Jcerity Medical Technology Co., Ltd) is a newly developed laryngeal mask that is specifically designed for endoscopy procedures in that it features an additional and separate groove at the back (Fig. 1). This device has shown promise in endoscopy at our gastrointestinal suite. Although there is a lack of research on the JEA, several studies are available on the LMA® Gastro™ airway (Teleflex Medical, Morrisville, North Carolina), a similar device featuring a 16 mm internal diameter gastroscopy channel^9^. A recent study revealed that the LMA® Gastro™ airway guarantees a high endoscope success rate and is easy to insert^10^, while another retrospective analysis demonstrated that the device is applicable for advanced endoscopic interventions in high-risk patients^11^. In addition, the LMA® Gastro™ airway device has been successfully applied in endoscopic retrograde cholangiopancreatography (ERCP), thereby validating its potential in treating high-risk patients who require airway management and have an American Society of Anesthesiologists (ASA) III-IV physical status^12–14^. Currently, endoscopic adjuncts, such as the variceal banding kit, can increase the diameter of the endoscope (15–16 mm), thereby posing a challenge for the LMA® Gastro™ airway's gastroscopy channel (16 mm), as it does not allow smooth insertion of the endoscope. On the other hand, the JEA features a larger internal diameter gastroscopy channel (Size 3: 18 × 20 mm, Size 4: 20 × 22 mm), which enables the smooth insertion of the variceal banding kit to allow EVL. Nonetheless, there is a lack of prospective trial data on the efficacy of the LMA^®^ Gastro™ airway and no data comparing the efficacies of JEA and ETT for high-risk patients undergoing complex endoscopic interventions such as EVL.

In this prospective controlled study, we evaluated the efficacy and safety of JEA in patients who received EVL.

## Methods

This prospective, randomized, parallel‑group study was approved by the Ethics Committee of the First Affiliated Hospital of Zhengzhou University (Zhengzhou, China) and was registered in the Chinese Clinical Trial Registry (ChiCTR), with registration number ChiCTR2000031892 (13/04/2020). The study design adhered to the guidelines of the International Conference on Harmonization Good Clinical Practice^15^. Written informed consent was obtained from all the participants prior to the enrollment of 164 patients with liver cirrhosis. We enrolled 164 liver cirrhosis patients who were scheduled to undergo elective EVL between May and September 2020. Patients with acute variceal hemorrhage were excluded. Age, sex, weight, height, BMI, ASA, Mallampati score, and comorbidities were recorded. A random sequence table was used to randomly assign participants to either the JEA group or the endotracheal intubation (ETT) group. To ensure the validity of the randomization process, duplicated random sequences were placed in opaque, sealed envelopes and reviewed by observers before anesthetics were administered. All data were collected by an independent unblinded observer in the perioperative period. All participants fasted from solid food and clear liquids for at least 8 and 2 h, respectively. Blood pressure was monitored noninvasively, while electrocardiographic images, pulse oximetry, and bispectral indexes (BIS) were routinely monitored in the operating room. All patients were given medications to prevent nausea and vomiting (0.06 mg/kg tropisetron) and esophageal peristalsis (papaverine). In the JEA group, after preoxygenation with 100% oxygen for 3 min, general anesthesia was induced using propofol (1–1.5 mg/kg), etomidate (0.1–0.15 mg/kg), and sufentanil (0.08–0.12 μg/kg). The patients in the ETT group received intravenous propofol (1–1.5 mg/kg), etomidate (0.1–0.15 mg/kg), sufentanil (0.08–0.12 μg/kg), remifentanil (0.4–1 μg/kg), and rocuronium (0.3 mg/kg). After the patient lost consciousness and the eyelash reflex was no longer observed, the JEA was inserted. Endotracheal intubation was used for manual ventilation until the modified alertness and sedation score (MOAA/S score) reached a maximum of three points or less^16^. An attending anesthesiologist inserted all airway devices. The size of the JEA was based on the patient's weight and the manufacturer’s instructions. The insertion of the airway device was considered successful when there was effective movement of the chest wall, with an airway leak pressure > 25–30 cmH_2_O and square-wave capnographic traces. Ventilation parameters were set at a tidal volume of 8 ml/kg and a rate of 8–15 bpm to keep end‑tidal carbon dioxide tension at 35–40 mmHg. Anesthesia was maintained with 1‑1.5% sevoflurane (Abbott Core Laboratory, Chicago) and its depth was monitored by the BIS, ranging from 40 to 60^17^, and remifentanil (4‑6 µg/kg/h) was continuously infused through a micropump (syringe infusion pump Model 500). All anesthetic regimens were discontinued upon completion of the last ligation. Once the patient regained consciousness and achieved sufficient spontaneous respiration, the JEA or ETT was removed. All endoscopic interventions were completed by qualified attending endoscopists with at least 5 years of experience.

### Observational data

We recorded the procedure success rate and the time taken for endoscopy insertion, EVL completion and airway device insertion. Blood pressure (BP), heart rate (HR), SpO_2_, and BIS were recorded at the following time points: baseline (the mean of the last 3 recordings before induction) (T0), the time of intubation (T1), 5, 10, and 15 min after intubation (T2, T3, T4), and the time of extubation (T5). The differences between the 2 BP values at different time points (△BP) and △HR were noted. The hemodynamic parameters were recorded every 3 min, and bradycardia (HR < 50 bpm), hypotension (systolic pressure < 90 mmHg) and hypertension (BP > 160/100 mmHg) were recorded as hemodynamic events.

The procedure duration was defined as the time between intubation and extubation of the gastroscope, whereas recovery time was defined as the time between anesthesia withdrawal and discharge from the recovery room. Extubation time was defined as the time between the end of anesthesia and the removal of the airway device. Adverse events or complications, including hypotension, hypertension (BP > 160/100 mmHg), bradycardia, sore throat, postoperative nausea or vomiting, urinary retention, poor muscle strength, and reflux, were recorded during and after endoscopic interventions. The attending endoscopist's satisfaction with advancing and operating the endoscope was assessed via VAS score: 0 = not satisfied, 10 = very satisfied.

### Sample size

A previous study of the LMA Gastro demonstrated an upper gastrointestinal endoscopy success rate of 99%^10^. We assumed that the success rates of EVL in the two groups would both be 96%. The predefined noninferiority margin was an absolute difference of 8% between groups for the primary endpoint. With a noninferiority margin of 8% on the relative scale, a power of 90%, and a one-sided alpha of 2.5%, the total sample size needed was 126. Assuming a dropout rate of 20%, a minimum of 79 patients were recruited for each group.

### Statistical analysis

Data analysis was performed using SPSS Statistics 19.0 software. Normally distributed data were presented as the means ± standard deviations (SD). Student's t test was utilized to compare measurements with normal distributions in a random block design. Nonnormally distributed continuous variables were analyzed using the Mann‒Whitney U test, while Tukey's multiple comparison test was used to compare measurements at different points within the same group. Comparisons between 2 groups were performed using the Bonferroni posttests, while proportions were compared using the Chi-square or Fisher's tests. A *P* value < 0.05 was statistically significant.

### Ethical approval and consent to participate

This trial was approved by the Clinical Research and Trial Ethics Committee of the First Affiliated Hospital of Zhengzhou University before its implementation. All the subjects who participated in the current trial signed informed consent forms.

## Results

**Figure 2:**
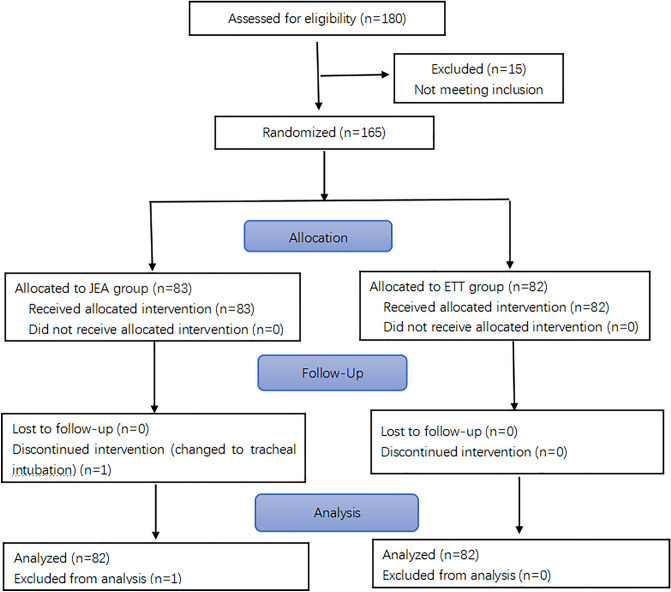
A schematic representation of participant recruitment process.

The participant recruitment process is shown in Fig. 2. A total of 180 patients were screened for eligibility, and 165 patients who met the inclusion criteria were selected. One patient in the JEA group had to be excluded because they required ETT conversion. Eventually, 164 patients were recruited, with 82 patients in each group (JEA and ETT). Table 1 shows that there were no significant differences in the patients’ characteristics between the two groups (*p* > 0.05).

**Table 1:** Characteristics of the patients who were recruited for the study.

	JEA (n = 82)	ETT (n = 82)	*P* value
Age (years)	53.5 ± 10.2	51.2 ± 10.2	0.150
Sex (male:female)	62:20	61:21	0.857
BMI	22.8 ± 3.4	23.6 ± 3.7	0.142
*ASA class*			> 0.999
III	80 (97.6)	79 (96.3)	
IV	2 (2.4)	3 (3.7)	
*Mallampati class*			0.690
I/II	65 (79.3)	68 (82.9)	
III	17 (20.7)	14 (17.1)	
*Comorbidities*			
Current smoker	32 (39.0)	29 (35.4)	0.628
History of drinking	32 (39.0)	32 (39.0)	> 0.999
Respiratory disease	1 (1.2)	2 (2.4)	> 0.999
Cardiovascular disease	7 (8.5)	8 (9.8)	0.786
Nervous system disease	1 (1.2)	1 (1.2)	> 0.999
Diabetes mellitus	6 (7.3)	10 (12.2)	0.292
Intravenous fluid volume before entry (ml)	125 (200)	153.5 (162.5)	0.517

Values are numbers (%), means ± SDs or median (IQR).

ASA, American Society of Anesthesiologists’ physical status.

### Airway insertion results

All airway devices were successfully inserted within three attempts. The insertion time for JEA was significantly shorter than that for ETT (*p* < 0.001) (Table 2).

**Table 2:** Procedural outcomes for patients in the JEA and ETT groups.

	JEA (n = 82)	ETT (n = 82)	*P* value
Airway device insertion time (s)	14.7 (7.3)	35 (14)	< 0.000
Endoscope Insertion attempts			
Successful insertion at first attempt	78 (95.1)	79 (96.3)	> 0.999
Successful insertion at second attempt	2 (2.4)	3 (3.7)	0.650
Successful insertion at third attempt	1 (1.2)	0 (0)	0.316
Endoscope Insertion time (s)	10 (5)	10 (5)	0.772
Procedure duration (min)	18 (10)	21 (15)	0.033
Extubation time (min)	7(3)	12 (6.3)	0.040
Anesthesia duration (min)	31 (14)	41 (24)	< 0.000
Recovery time (min)	18.5 (6)	26 (10.3)	< 0.000
Endoscopist’s satisfaction	7.9 ± 0.2	7.9 ± 0.3	0.390
Anesthesia costs (RMB, YUAN)	2329 (282)	2686 (371)	< 0.000
Hospital stay (d)	7 (5)	9 (5.2)	0.048

Data presented are numbers (%), means ± SDs or median (IQR).

Extubation time denotes the time between anesthesia withdrawal and removal of the airway device.

### Endoscopy results

The success rate of EVL with the JEA was 98.8% (95% CI 96.6–100), with success defined as no more than three attempts. On the other hand, the success rate of EVL with the ETT was 100% (95% CI 100–100). The difference in success rates between the JEA and ETT groups was −1.2% (95% CI −3.4%, 0.9%). Our findings suggested that JEA was noninferior to ETT in patients who underwent elective EVL, with the noninferiority limit set to 8%. Both groups demonstrated a comparable first-attempt endoscopy success rate (95.1% vs. 96.3%, *p* > 0.999), and we observed no significant differences (*p* > 0.05) in their mean insertion times. One attempt to place the JEA was defined as unsuccessful because the endoscope was not successfully inserted after three attempts. Nonetheless, there was no difference in the attending endoscopist's satisfaction with advancing and operating the endoscope between the two groups (Table 2).

### Parameters related to anesthesia

The JEA group exhibited shorter extubation time, shorter recovery time, procedure duration, and anesthesia duration compared to the ETT group (*p* < 0.05). Additionally, our analysis revealed that the use of JEA resulted in a significant reduction in anesthesia costs (*p* < 0.001) and hospital stays (*p* < 0.05) (Table 2).

### Hemodynamic variables

**Figure 3:**
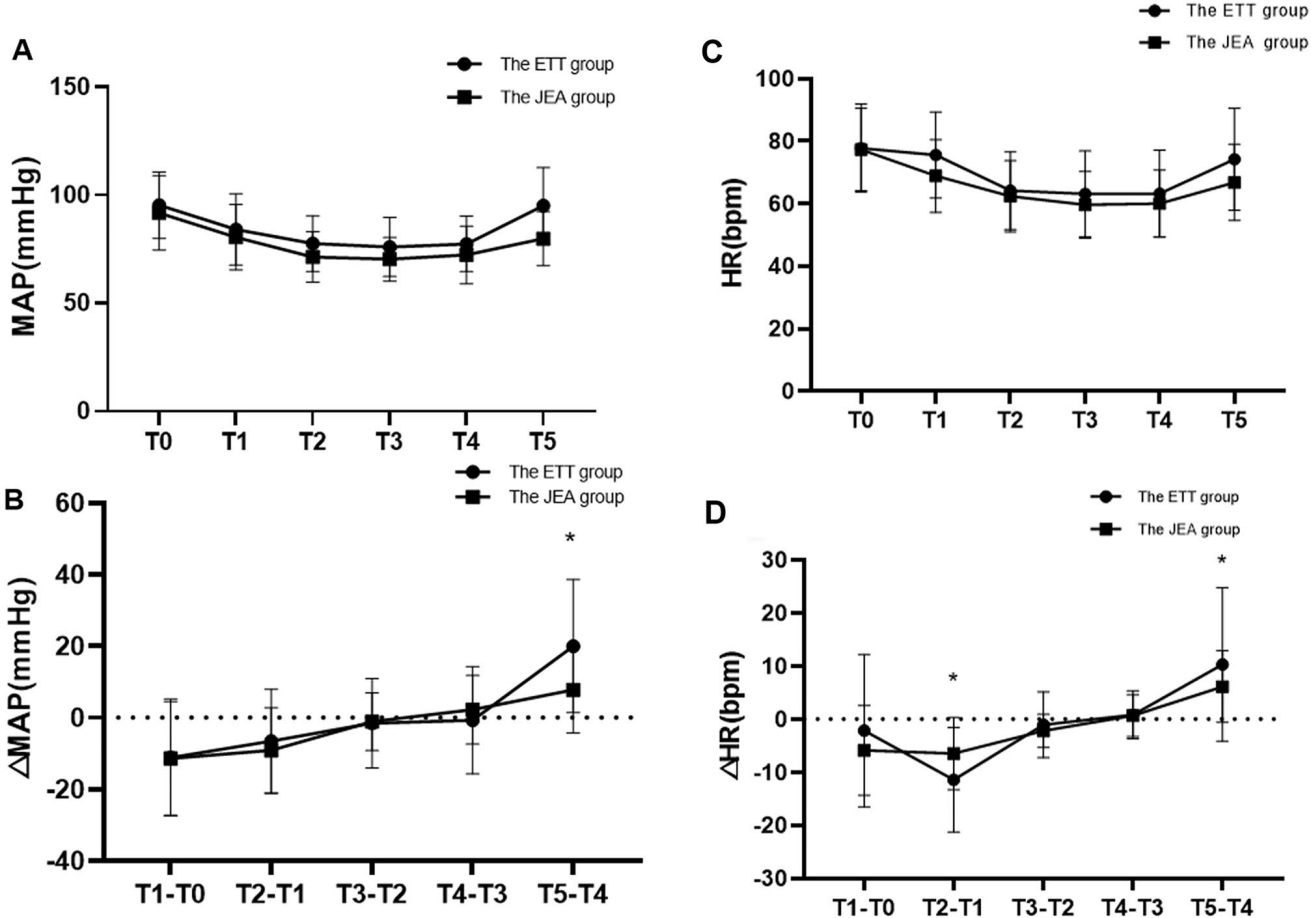
Comparisons in MAP and HR differences between the ETT vs the JEA groups at different timepoints. (**A**) MAP at different timepoints in two groups; (**B**) Comparisons in MAP difference between the ETT vs the JEA groups at different timepoints; (**C**) HR at different timepoints in two groups; (**D**) Comparisons in MAP difference between the ETT vs the JEA groups at different timepoints; Data represents means ± SD. *p < 0.05.

The induction of anesthesia led to a significant decrease in mean arterial pressure (MAP) and heart rate (HR) in all participants, with these parameters gradually increasing between time points T4 and T5 (Fig. 3A,C). Notably, MAP fluctuations in the JEA group were significantly smaller than those in the ETT group at time points T4-T5 (*p* < 0.001), as presented in Fig. 3B. Furthermore, HR fluctuations in the JEA group were significantly smaller than those in the ETT group at time points T1-T2 (*p* < 0.05) and T4-T5 (*p* < 0.05, Fig. 3D).

### Complications

We observed a lower incidence of sore throat in the JEA group than in the ETT group, although the difference between them was not statistically significant (*p* > 0.05). Our analysis revealed no significant differences between the two groups in the incidence of postoperative nausea, vomiting, or urinary retention (*p* > 0.05). Moreover, we observed no perioperative bleeding or reflux cases in either group. However, a higher number of patients in the ETT group exhibited a blood pressure reading higher than 160/100 mmHg during extubation (*p* < 0.05), and a higher proportion of patients in the ETT group exhibited poor muscle strength after extubation (*p* < 0.05) (Table 3).

**Table 3:** Patient complications associated with both devices.

Complication	JEA (n = 82)	ETT (n = 82)	*P* value
Vasoactive drugs use	12 (14.6)	15 (18.3)	0.528
BP > 160/100 mmHg	1 (1.2)	9 (10.9)	0.018
Sore throat	6 (7.3)	8 (9.8)	0.576
Postoperative nausea or vomiting	10 (12.2)	12 (14.6)	0.647
Urinary retention	0 (0)	1 (1.2)	> 0.999
Poor muscle strength	0 (0)	7 (8.5)	0.014
Reflux	0 (0)	0 (0)	> 0.999

Data presented are numbers (%).

## Discussion

**Figure 4:**
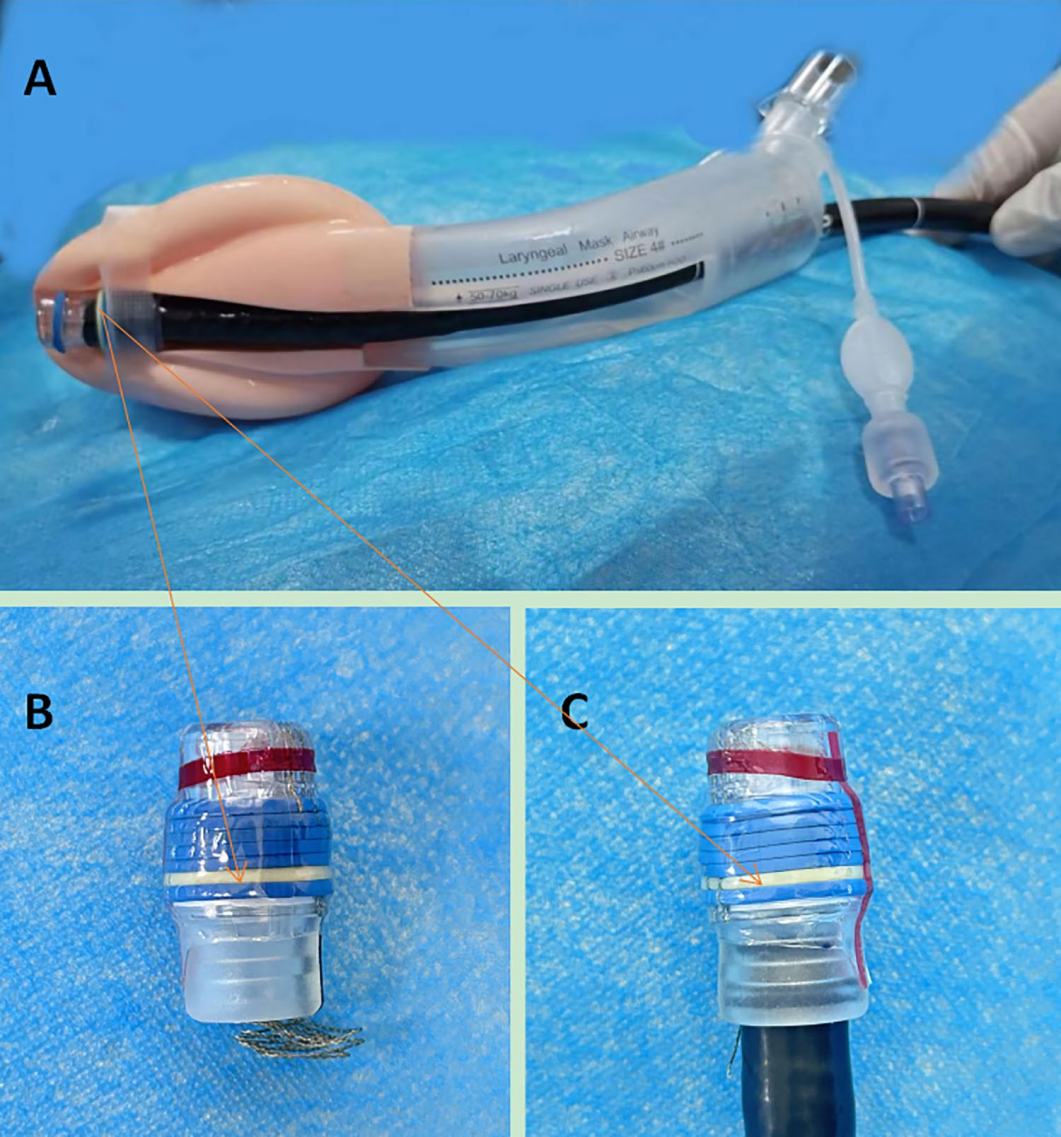
Technical parameters of JEA and variceal banding kit, which was shot and edited by Lu Li. (**A**) Gastroduodenoscope with the variceal banding kit passing through the gastric channel of a JEA; (**B**) The outer diameter of variceal banding kit (Max 15.9 mm); (**C**) The diameter of the gastroduodenoscope with the variceal banding kit (16 mm).

This study provides evidence that the JEA is a viable option for airway management during EVL procedures under general anesthesia. In this respect, we observed that the success rate of EVL with the JEA was 98.8% (95% CI 96.6–100) and that first-attempt success rate was 96%. These results were consistent with previous studies on the LMA® Gastro™ airway (one similar airway device) for esophagogastroduodenoscopy (EGD)/ERCP^10,18^. It should be noted that the variceal banding kit has a larger outer diameter (approximately 15–16 mm, Fig. 4) than gastroscopes used for EGDs (4.9–12.8 mm) and duodenoscopes (7.5–12.1 mm)^19^. The maximum diameter of an endoscope that could successfully pass through the endoscopy channel of the LMA® Gastro™ airway was 14 mm. Thus, an endoscope with a variceal banding kit could not pass through the LMA® Gastro™ airway. In contrast, the JEA has a larger and adjustable endoscope channel, thus facilitating endoscope advancement and operation (Table 4). In addition, the variceal banding kit increases the length of the endoscope tip, reducing endoscopic vision by 30%^20^, which could increase the difficulty of endoscope insertion. Nevertheless, the JEA’s endoscopy channel ends at the cuff's distal tip to align with the esophageal entrance, thereby facilitating endoscope insertion. As such, our endoscopists obtained equal or better access to the gastrointestinal tract with the JEA device as with the ETT. Furthermore, the JEA group had shorter procedure durations. No significant difference was observed in the endoscopist's satisfaction with endoscope advancement or operation between the JEA and ETT groups, indicating that the JEA could be a valuable alternative to the LMA® Gastro™ airway and endotracheal intubation for EVL.

**Table 4:** List of physical characteristics and specifications for Jcerity endoscoper airway and LMA Gastro airway.

Specification	Jcerity endoscoper airway (JEA)	LMA Gastro airway
*Manufacturer*		
Name	Jcerity Medical	Teleflex Medical
Address	Zhejiang Jcerity Medical Corp, China	Morrisville, North Carolina
Website	www.jenstonmed.com	www.teleflex.com
*Laryngeal mask*		
Available sizes, based on weight (kg)	Single use	Single use
	3(30–50);4(50-70 kg)	3(30–50);4(50–70);5(70-100 kg)
Material cuff/tube	Medical silicone	Medical silicone
Inflation device	Inflatable indicator airbag	Pressure indicating device
*Laryngeal mask channels*		
Functional separation	Yes	Yes
*Position of orifices*		
-Airway tube	On the left side of device	On the left side of device
-Endoscope tube	Middle	Middle
*Laryngeal mask design*		
Design of cuff	Enlarged air-inflatable cuff, high airway seal cuff	Enlarged air-inflatable cuff, high airway seal cuff, reinforced tip
Airway tube–anatomically shaped oval tube	Yes	Yes
Airway tube–flexible-curved	Yes	Yes
Internal diameter airway channel(mm)	8(all sizes)	
Original main tube curvature	116°	
Colour of cuff/tube	Pink/transparent	White/blue
Built-in bite block	Yes	Yes
Indicator on tube of insertion depth	Yes	Yes
Phthalates (DHEP) free	Yes	Yes
Inflation valve, Luer cone, ISO 594–1	Yes	Yes
Maximal cuff pressure (cm H_2_O)	60	60
*Laryngeal Mask as endoscopy*		
Design of endoscope tube	Semi-enclosed	Enclosed
Cross-section of endoscope tube	Elliptical	Circular
Internal diameter endoscope channel (mm)	3(18 × 20);4(20 × 22)	16 (all sizes)
Maximum size for endoscope (mm)		14 (all sizes)

In this study, there was no bleeding, reflux or other severe complications in the JEA group. Due to the high risks of perioperative bleeding and reflux^6^ associated with EVL, general anesthesia with ETT is often utilized to prevent aspiration. However, studies have shown that patients at high risk of aspiration have similar incidences of regurgitation when using either ETT or a supraglottic airway (SGA) with a gastric suction channel ^21,22^. The JEA has a crucial advantage over SGA in terms of airway protection as it enables direct suctioning under visualization during gastroscopy.

Nonoperating anesthesia is recommended to be efficient, timely, and safe^23^. The overall success rates achieved by the JEA and ETT devices in this study were equal to or greater than those reported in previous studies on the use of other supraglottic airway tools^24^. In addition, the JEA had a significantly shorter insertion time (14.7 (7.3) s) than the ETT and other supraglottic airway tools, such as the laryngeal mask airway supreme (LMS) (48.8 ± 45.6 s) and i-gel (34.7 ± 64.4 s)^17^. This finding can be attributed to the JEA device's soft material and flexibility, making it more easily insertable and practical in clinical settings. This flexibility is attributed to the groove at the back of the LMA, making it slightly thinner. The high success rate of JEA insertion may also be attributed to the patient's lateral position, which allows for a larger oropharyngeal cavity space and the tongue to fall out of the path of the airway device^25^ These reasons may contribute to the high level of endoscopist's satisfaction with JEA placement^18^.

Our study showed that JEA placement resulted in significantly shorter anesthesia durations, extubation times, and recovery times than ETT placement, potentially improving patient turnover efficiency. Furthermore, we observed more hemodynamic stability, lower anesthesia costs, and shorter hospital stays in the JEA group, which may be due to the LMA reducing the dosage of anesthetics needed, which subsequently shortens the recovery time following endoscopic procedures. In addition, muscle relaxants that may affect recovery time are not required when LMA is used. It is now understood that anesthetics can trigger vasodilation and negative inotropic effects, thereby affecting blood pressure and heart rate. Use of the JEA during anesthesia induction can potentially lead to several benefits, such as reducing the volume of anesthetic agents needed to maintain anesthesia, thus resulting in faster extubation, a shorter recovery time, better hemodynamic stability, and potentially lower anesthesia costs. The relatively high cost of the JEA device, which is approximately 310 yuan (equivalent to $45), could potentially be compensated by the decreased usage of anesthetic and vasoactive drugs and the elimination of the need for disposable laryngoscope lenses and muscle relaxant antagonists. Ultimately, the total anesthesia costs in the JEA group were lower than those in the ETT group. Furthermore, JEA could ease the transition between sleep and wakefulness in the EVL without stimulating the airway. Significantly lower rates of coughing and cardiovascular reactions have been reported during LMA® Gastro™ airway insertion and removal in endoscopic submucosal dissection (ESD) than during ETT insertion and removal^26^. In the present study, extubation of the JEA caused minimal fluctuations in both the MAP and HR, without causing severe hypertension (> 160/100 mmHg). Collectively, these findings substantiate the efficacy and safety of the JEA.

Patients with liver cirrhosis often have multiple serious health conditions in addition to poor liver function. Therefore, reducing the volume of anesthetics used can help reduce the metabolic burden on the liver, prevent drug accumulation, and improve postoperative recovery while minimizing the incidence of complications. In this study, we observed a shorter recovery time in the JEA group but no significant differences in the incidence of complications between the two groups. Additionally, neither group experienced any instances of aspiration during the procedure. Deep anesthesia may be beneficial for reducing body movement during EVL and decreasing the risk of bleeding, thereby improving the procedure's success rate. Previous studies suggest that propofol-based TIVA can improve the adenoma detection rate^27,28^, while deep anesthesia using a LMA Gastro may improve common bile duct cannulation and stent placement^29^. The JEA improves airway access through a dedicated gastroscopy channel, thereby improving airway control and preventing hypoxia while avoiding the need for intubation in deep anesthesia during endoscopy.

Patients undergoing endoscopic variceal sclerotherapy were excluded from our study because there was a risk of glue sticking to the JEA.

Our study suggests that the JEA may be a safe alternative for patients undergoing EVL. Before final recommendations are made, it is necessary to conduct large multicenter trials to assess the impact of JEA on liver function and the clinical outcomes of EVL.

In conclusion, this study substantiated that JEA placement is effective and safe in EVL procedures, thus offering benefits such as a faster operating room turnover, better maintenance of hemodynamic stability, reduced patient anesthesia costs, and shorter hospital stays.

## Data Availability

The datasets used during the present study are available from the corresponding author upon reasonable request.

## Abbreviations

EVL: Endoscopic esophageal variceal ligation
JEA: Jcerity endoscoper airway
LMA: Laryngeal mask airway
ETT: Endotracheal tube
ERCP: Cholangiopancreatography
BP: Blood pressure
HR: Heart rate
EGD: Esophagogastroduodenoscopy
SGA: Supraglottic airway
